# Cytotoxic activity of the novel small molecule AKT inhibitor SC66 in hepatocellular carcinoma cells

**DOI:** 10.18632/oncotarget.2738

**Published:** 2014-12-19

**Authors:** Antonella Cusimano, Roberto Puleio, Natale D'Alessandro, Guido R. Loria, James A. McCubrey, Giuseppe Montalto, Melchiorre Cervello

**Affiliations:** ^1^ Institute of Biomedicine and Molecular Immunology “Alberto Monroy”, National Research Council, Palermo, Italy; ^2^ Istituto Zooprofilattico Sperimentale della Sicilia “A. Mirri”, Area Diagnostica Specialistica, Laboratorio di Istopatologia ed Immunoistochimica, Palermo, Italy; ^3^ Dipartimento di Scienze per la Promozione della Salute e Materno Infantile “G. D'Alessandro”, Università di Palermo, Palermo, Italy; ^4^ Department of Microbiology and Immunology, Brody School of Medicine at East Carolina University, Greenville, NC, USA; ^5^ Biomedical Department of Internal Medicine and Specialties, University of Palermo, Palermo, Italy

**Keywords:** HCC, AKT, mTOR, SC66, anoikis

## Abstract

Hepatocellular carcinoma (HCC) is characterized by limited response to current drug therapies. Here, we report that SC66, a novel AKT inhibitor, reduced cell viability in a dose- and time-dependent manner, inhibited colony formation and induced apoptosis in HCC cells. SC66 treatment led to a reduction in total and phospho-AKT levels. This was associated with alterations in cytoskeleton organization, a reduction in expression levels of E-cadherin, β-catenin and phospho-FAK, together with up-regulation of Snail protein levels. All these alterations were coupled with anoikis cell death induction. In addition, SC66 induced the production of reactive oxygen species (ROS) and DNA damage. Pre-treatment with the ROS scavenger N-Acetyl-cysteine (NAC) prevented SC66-induced cell growth inhibition and anoikis. SC66 significantly potentiated the effects of both conventional chemotherapeutic and targeted agents, doxorubicin and everolimus, respectively. *In vivo*, SC66 inhibited tumor growth of Hep3B cells in xenograft models, with a similar mechanism observed in the *in vitro* model. Taken together, these data indicate that the AKT inhibitor SC66 had antitumor effects on HCC cells. This was mediated by ROS production, induction of anoikis-mediated cell death and inhibition of the AKT cell survival pathway. Our results provide a rational basis for the use of SC66 in HCC treatment.

## INTRODUCTION

Hepatocellular carcinoma (HCC) is the fifth most common cancer worldwide, characterized by an increasing incidence and a poor prognosis [1–5]. HCC is largely asymptomatic until it is in the advanced state when the treatments available are often unsuccessful. The standard treatments are surgical resection and liver transplantation, but they are indicated only in specific conditions, however both are rarely successful. Other treatments, such as chemoablation, focused ultrasound and radiation also rarely lead to complete recovery [5]. Standard cancer drugs such as doxorubicin, cisplatin, and 5-fluorouracil have very limited efficacy [5].

Today, sorafenib, a Raf kinase inhibitor approved by the FDA and EMEA for the treatment of patients with advanced and unresectable HCC, is the only systemic therapy which improves survival in HCC patients [6]. Although sorafenib improves prognosis in advanced HCC, response to sorafenib remains low and the median overall survival is only extended by a few months [6–10].

The development of new specific therapies is necessary. However, recent knowledge of the oncogenic processes and signaling pathways that regulate tumor cell proliferation, differentiation, angiogenesis, invasion and metastasis has led to the identification of several potential molecular targets and to the development of specific targeted molecular therapies.

The PI3K/AKT/mTOR signaling pathway is involved in various cellular processes such as cell proliferation, migration, survival and angiogenesis. In PI3K/AKT/mTOR signaling, activated receptors trigger the activation of PI3K and the conversion (by its catalytic domain), of phosphatidylinositol (3,4)-bisphosphate (PIP2) lipid to phosphatidylinositol (3,4,5)-trisphosphate (PIP3). AKT binds to PIP3 at the plasma membrane, allowing PDK1 to phosphorylate AKT to T308, leading to partial AKT activation [11, 12]. Phosphorylation of AKT at S473, either by mTOR [13] or by DNA-PK [14], stimulates full AKT activity. Full activation of AKT leads to additional substrate-specific phosphorylation events in both the cytoplasm and nucleus [15]. Fully-active AKT mediates numerous cellular functions including angiogenesis, metabolism, growth, proliferation, survival, protein synthesis, transcription and apoptosis.

Phosphorylation of AKT at S473 has been detected in up to 71% of HCC samples and associated with the invasion, metastasis, and vascularization of HCC [16].

Activated AKT positively modulates mammalian target of rapamycin (mTOR) function. mTOR phosphorylates components of the protein synthesis machinery, such as the serine-threonine kinase p70^S6^ (40S ribosomal protein kinase) and the translation repressor eukaryotic initiation factor 4E-binding protein-1 (4E-BP1), both regulating the translation of important factors involved in cell proliferation (such as c-myc, cyclic D1 and pRb) and angiogenesis (such as HIF1-α). Aberrant mTOR signaling has also been detected in up to 48% of HCC cases, and a correlation between poor outcome and mTOR signaling activation has been shown [17, 18]. These results make the AKT/mTOR signaling a promising target for new therapies in HCC [19–22].

However, the clinical relevance of the AKT/mTOR pathway as a key target in HCC and its therapeutic potential as a target for therapy remain to be elucidated. Recently, the novel AKT inhibitor SC66 has been demonstrated to promote cell death in cervical cancer through disruption of mTOR signaling [23]. SC66 is an allosteric inhibitor which displays a dual-inhibitory function toward AKT activity: i) deactivation by facilitated ubiquitination, and ii) inhibition by directly interfering with the pleckstrin homology (PH) domain binding to PIP3 [24].

In this study, we analyzed the antitumor activity of SC66 in HCC cells *in vitro* and *in vivo*. We demonstrated that treatment with SC66 induced cell growth inhibition, inhibition of colony formation and anoikis through ROS production. SC66 significantly inhibited cell viability in combination with doxorubicin and everolimus. SC66 was also effective in *in vivo* xenograft-bearing mice where it displays significant tumor growth reduction. These findings suggest that SC66 might represent a promising new therapeutic drug for HCC treatment.

## RESULTS

### SC66 inhibits cell viability and colony forming capacity of HCC cells

**Figure 1:**
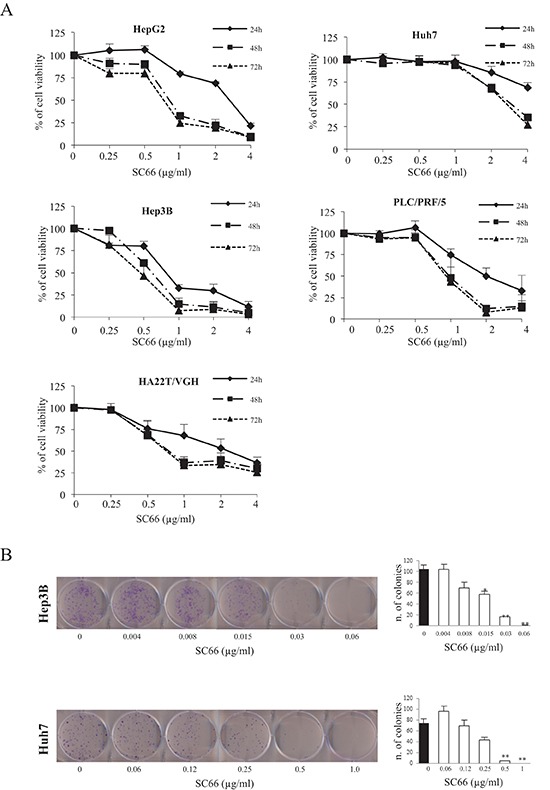
SC66 is cytotoxic to HCC cell lines **(A)** Cell viability in each HCC cell line was assessed by MTS assays. Cells were treated with increasing concentrations of SC66 for 24, 48 and 72 hours. Data are expressed as the percentage of control cells and are the means ± SD of three separate experiments, each of which was performed in triplicate. **(B)** Representative images of clonogenic assay after treatment with SC66. Hep3B and Huh7 cells were plated overnight and exposed to SC66 at the indicated concentrations for 48 hours. After treatment each well was washed and the experiment continued for 14 days in the absence of drugs. Surviving colonies were stained (left panel) and counted (right panel). Data are expressed as a numbers of colonies and are the means ± SD of two separate experiments, each of which was performed in duplicate. *p < 0.05, **p < 0.001 versus control vehicle alone.

To investigate the effects of SC66 on HCC cell viability, HepG2, Huh7, Hep3B, PLC/PRF/5 and HA22T/VGH cell lines were incubated with increasing concentrations of SC66 and cell viability was analyzed after 24, 48 and 72 hours. Our results demonstrated that treatment with SC66 reduced cell viability in a dose- and time-dependent manner (Figure 1A). Each cell line had a different sensitivity to the drug, as evidenced by the IC_50_ values shown in Table 1. HepG2, HA22T/VGH and PLC/PRF/5 cells had similar IC_50_ values of approximately 0.85 and 0.75 μg/ml at 48 and 72 hours, respectively. The most resistant cell line was Huh7, which showed an IC_50_ of 3.1 and 2.8 μg/ml at 48 and 72 hours respectively, while the Hep3B cell line was found to be the most sensitive, with an IC_50_ of 0.75 and 0.5 μg/ml at 48 and 72 hours, respectively. For example, at 24 hours the highest dose tested (4 μg/ml) inhibited Huh7 cell viability by almost 30% and Hep3B cell viability by almost 90% (Figure 1A), therefore we selected these two cell lines for all further experiments.

**Table 1:** IC_50_ (μg/ml) values after treatment with SC66

	HepG2	Huh7	Hep3B	PLC/PRF/5	HA22T/VGH
**24h**	2.27	>4	0.82	2	2.35
**48h**	0.86	3.09	0.75	0.97	0.8
**72h**	0.77	2.85	0.47	0.92	0.75

Next, we performed a colony forming assay (Figure 1B), which mimics the clonogenic survival of tumor cells in a solid tumor environment. SC66 displayed a strong inhibition of colony forming capacity, and this type of assay also confirmed that Hep3B cells have a higher sensitivity to the drug than Huh7 cells (Figure 1B).

### SC66 induces apoptosis

**Figure 2:**
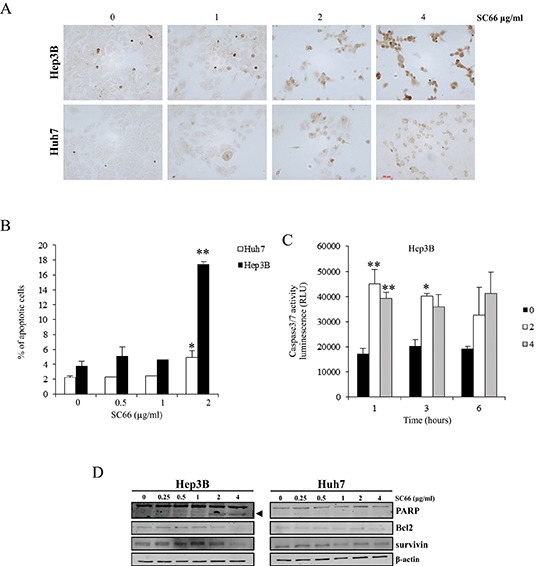
SC66 induces apoptosis in HCC cell lines **(A)** Detection of apoptosis by TUNEL assay as described in the Materials and Methods section. Photomicrographs of Hep3B and Huh7 cells treated for 24 hours with increasing concentrations of SC66. **(B)** Cell death was determined by flow cytometry analysis of DNA stained with propidium iodide and evaluation of the percentage of events accumulating in the pre-G0-G1 position. Data are expressed as percentages of apoptotic cells and are the mean ± SD of two separate experiments. *p < 0.05, **p < 0.001 versus control. **(C)** The levels of caspase activity in the cells were measured by the Caspase-Glo® 3/7 assays after treatment with 0, 2, 4 μg/ml of SC66. Data are expressed as relative luminescence units (RLU) and are the means ± SD of three separate experiments, each of which was performed in duplicate. *p < 0.05, **p < 0.001, versus control. **(D)** PARP cleavage induction and levels of survivin, and Bcl2 proteins were analyzed by Western blot. The data shown represent three independent experiments with comparable outcomes. The arrowhead indicates the 85 kDa form of PARP.

To determine whether the decrease in cell viability was related to apoptosis induction, TUNEL assays were performed in Hep3B and Huh7 cells treated with 1, 2 and 4 μg/ml of SC66 for 24 hours. As shown in Figure 2A, in Hep3B cells the number of TUNEL-positive cells increased with increasing concentrations of SC66, whereas in Huh7 cells very few light brown-colored cells were observed only after treatment with 4 μg/ml SC66.

Apoptosis was also quantified by flow cytometry analysis of DNA stained with propidium iodide and by determining the percentage of events accumulating in the subG1 position (Figure 2B). Treatment with 2 μg/ml SC66 increased apoptotic Hep3B cells to 17.5% ± 0.3 compared to control, whereas the percentage of apoptotic cells was only 4.5% ± 0.8 in the more resistant Huh7 cells. Consistent with the apoptosis detected in Hep3B cells, the kinetics of caspase-3/7 activity measured by fluorometric caspase-3/7 assay showed early activation of caspase-3/7 starting as little as 1 hour after treatment (Figure 2C). Caspase activity after 1, 3 and 6 hours of SC66 exposure was significantly higher in Hep3B cells treated with SC66 2 and 4 μg/ml than in Hep3B cells treated with vehicle alone (*p* < 0.001). In Huh7 cells, we observed a 1.3 fold increase in caspase activity only at 24 hours and only with the highest drug dose (data not shown).

Finally, Western blot analysis of protein extracted from Hep3B cells after treatment for 24 hours with 2 and 4 μg/ml SC66 showed a dose-response cleavage of PARP and a decrease in anti-apoptotic proteins Bcl2 and survivin (Figure 2D). In Huh7 cells after SC66 treatment the same proteins maintained the baseline levels observed in untreated cells (Figure 2D).

All these analyses highlight that the decrease in cell viability observed after SC66 treatment was due to apoptosis induction.

### SC66 affects AKT/mTOR signaling in HCC cell lines

**Figure 3:**
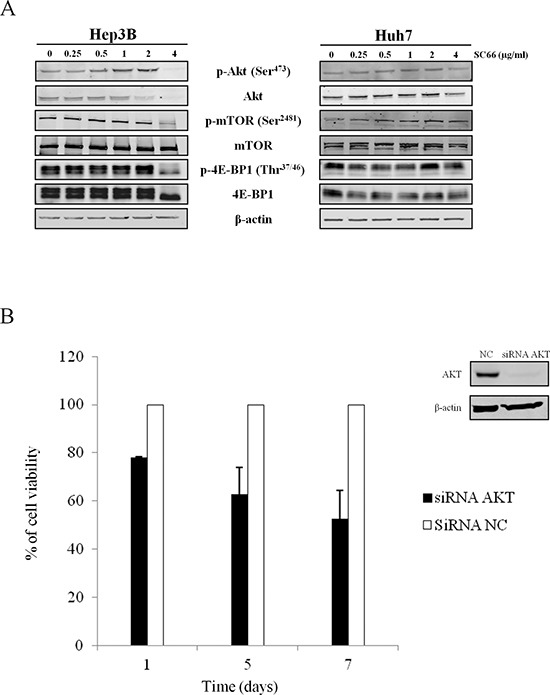
Effects of SC66 on the phosphorylation status of critical components of the cellular signaling pathway **(A)** Western blot analysis of phospho- and total-AKT, phospho- and total-mTOR, phospho- and total- 4E-BP1 in a dose-dependent treatment. β-actin served as a loading control. The data shown represent three independent experiments with comparable outcomes. **(B)** (left) Cell viability of Hep3B cells transfected with siRNA AKT and siRNA NC was assessed by MTS assay. Data are expressed as the percentage of control cells and are the means ± SD of three separate experiments, each of which was performed in triplicate. (right) Western blot of target protein in cells transfected with siRNA AKT and siRNA NC.

As SC66 is a novel allosteric AKT inhibitor which directly interacts with AKT and facilitates its ubiquitination and deactivation [24], we investigated whether the cytotoxic effects of SC66 were related to changes in AKT signaling. Western blot analysis was performed after SC66 treatment. As observed in Figure 3, the dose-response curve showed a slight increase in phospho-AKT expression levels in the presence of SC66 at concentrations of 1 and 2 μg/ml, whereas phospho-AKT expression levels dramatically decreased after treatment with 4 μg/ml SC66 (Figure 3A). Analysis of total AKT protein levels revealed a decrease in protein already at a dose of 2 μg/ml SC66 in Hep3B cells. In Huh7 cells, we observed a slight effect on AKT expression levels only after treatment with 4 μg/ml SC66 (Figure 3).

We also examined phosphorylation levels of mTOR, a well-established down-stream target of activated AKT. SC66 treatment resulted in a significant fall in mTOR phosphorylation in Hep3B cells (Figure 3A). To confirm the modulation of mTOR signaling, we evaluated the phosphorylation levels of 4E-BP1 protein, a mTOR downstream target. Treatment with 4 μg/ml SC66 reduced phospho-4E-BP1 (p-4E-BP1) and of total 4E-BP1 protein levels in Hep3B cells (Figure 3A). In Huh7 cells, we observed a slight effect on both p-4E-BP1 and total 4E-BP1 expression levels after treatment with SC66 (Figure 3).

### Silencing of AKT partially overlaps the effects of SC66

To evaluate how dependent the effects of SC66 were on target AKT expression, we silenced the *AKT* gene with a specific small interference RNA (siRNA). Although, after silencing of AKT by siRNA, AKT protein expression was reduced by 80% after 72 hours, AKT silencing reduced cell viability by almost 50% only after 7 days (Figure 3B). At the morphological level, no morphological features of apoptosis were observed in cells transfected with AKT siRNA (not shown). Therefore, the effect observed was less strong than the one observed using the pharmacological approach with SC66. These results suggest that the effects of SC66 are only partially AKT-dependent.

### SC66 induces anoikis

**Figure 4:**
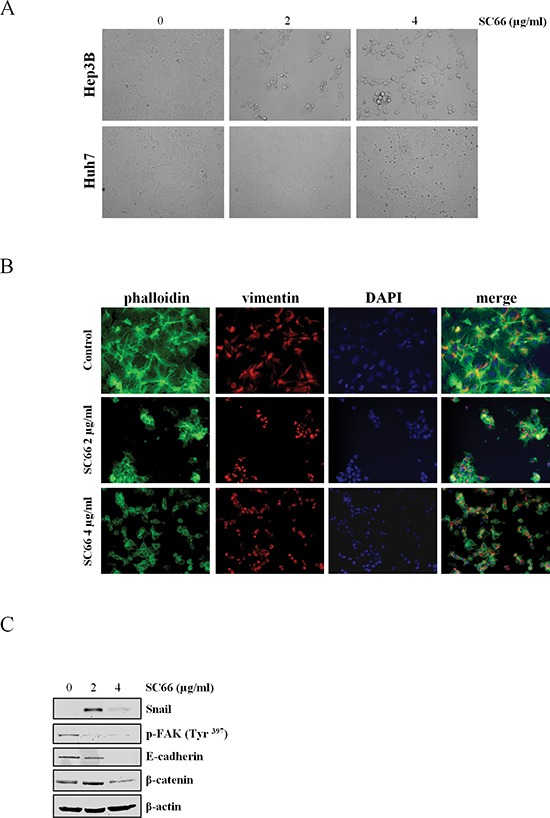
SC66 induces anoikis **(A)** Photomicrographs showing the morphology of Hep3B and Huh7 cells after SC66 treatment. **(B)** Immunofluorescence staining of DAPI (blue), vimentin (red) and phalloidin (green) in Hep3B cells treated with SC66 at 2 and 4 μg/ml for 24 hours. Morphological changes in cytoskeleton organization are visible after treatments. **(C)** Western blot analysis demostrating induction of Snail and decreases in phospho-FAK, E-cadherin and β-catenin protein levels. The data shown represent two independent experiments with comparable outcomes. β-actin immunolabeling was performed as a loading control.

The above results prompted us to investigate whether any additional mechanism(s) that could be responsible for the effects of SC66 activity on cell viability. Microscopic examination revealed that when exposed to increased concentrations of the drug the more sensitive Hep3B cells became rounded, slightly attached to each other, similar to beads on a string, and detached from the substrate, losing contact with extracellular matrix molecules (ECM) (Figure 4A). No evident effects were visible in Huh7 cells treated at the same drug doses and at the same treatment times (Figure 4A). Following these observations we hypothesized that in Hep3B cells SC66 might induce anoikis, a form of programmed cell death resulting from the loss of cell adhesion to the ECM [25–31]. Therefore, certain markers of anoikis, such as cytoskeleton and cell membrane-associated proteins, were analyzed. To observe the distribution of cytoskeleton microfilaments after treatment with SC66, Hep3B cells were stained with both phalloidin, which specifically stains actin filament, and with anti-vimentin antibody. As shown in Figure 4B, changes in distribution of actin and vimentin occurred after drug treatment, and in particular the latter seemed to slightly increase and accumulate in the perinuclear zone (Figure 4B). Western blot analysis also showed a reduction in E-cadherin expression levels and an induction of Snail, the negative regulator transcription factor of *E-cadherin (CDH1)* gene (Figure 4C) [32].

We also evaluated β-catenin protein levels as it is a component of adhesion complexes. As shown in Figure 4C β-catenin levels decreased after SC66 treatment.

The effects of SC66 on FAK phosphorylation levels were also evaluated. Treatment with SC66 clearly reduced the phosphorylated form of FAK (Figure 4B). In addition, cells floating after SC66 exposure were not able to re-adhere to the plate after the inhibitor was removed and washed cells were plated again, suggesting that the death program is irreversible (data not shown).

### SC66 induces ROS production in Hep3B cells

Many compounds may sensitize cancer cells to anoikis through ROS generation [33–36]. In addition, ROS have also been reported to be important mediators of anoikis and also to be related to caspase activation [34, 36]. Consequently, we investigated whether SC66 antitumor activities in HCC cells were initiated through ROS generation.

**Figure 5:**
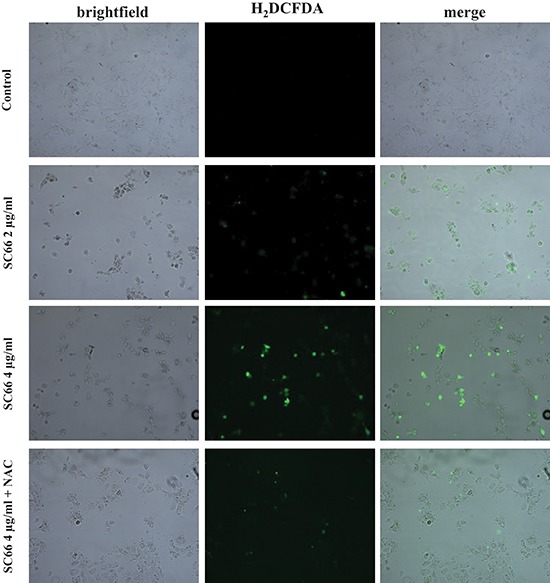
SC66 induces ROS production Hep3B cells were untreated or treated with the indicated concentrations of SC66 for 24 hours with or without the presence of the antioxidant NAC (2 mM). The cells were incubated with the fluorescent probe H_2_DCFDA and fluorescence was analyzed by fluorescence microscopy. Photomicrographs show images in bright field of Hep3B cells and in fluorescence (green) for the H_2_DCFDA probe.

Using the cell-permeable fluorescent probe H_2_DCFDA, we found that SC66 treatment induced intracellular ROS production in Hep3B cells in a dose-dependent manner (Figure 5). Treatment with antioxidants, such as N-Acetyl-cysteine (NAC), a ROS scavenger, should abrogate ROS effects. For this purpose cells were pretreated with 2 mM NAC for 2 hours before being treated with SC66. As shown in Figure 5 the intensity of the fluorescence probe increased in the presence of SC66, and decreased when cells were treated with SC66 in the presence of NAC.

### Treatment with NAC reverts the cytotoxic effects of SC66 in Hep3B cells

**Figure 6:**
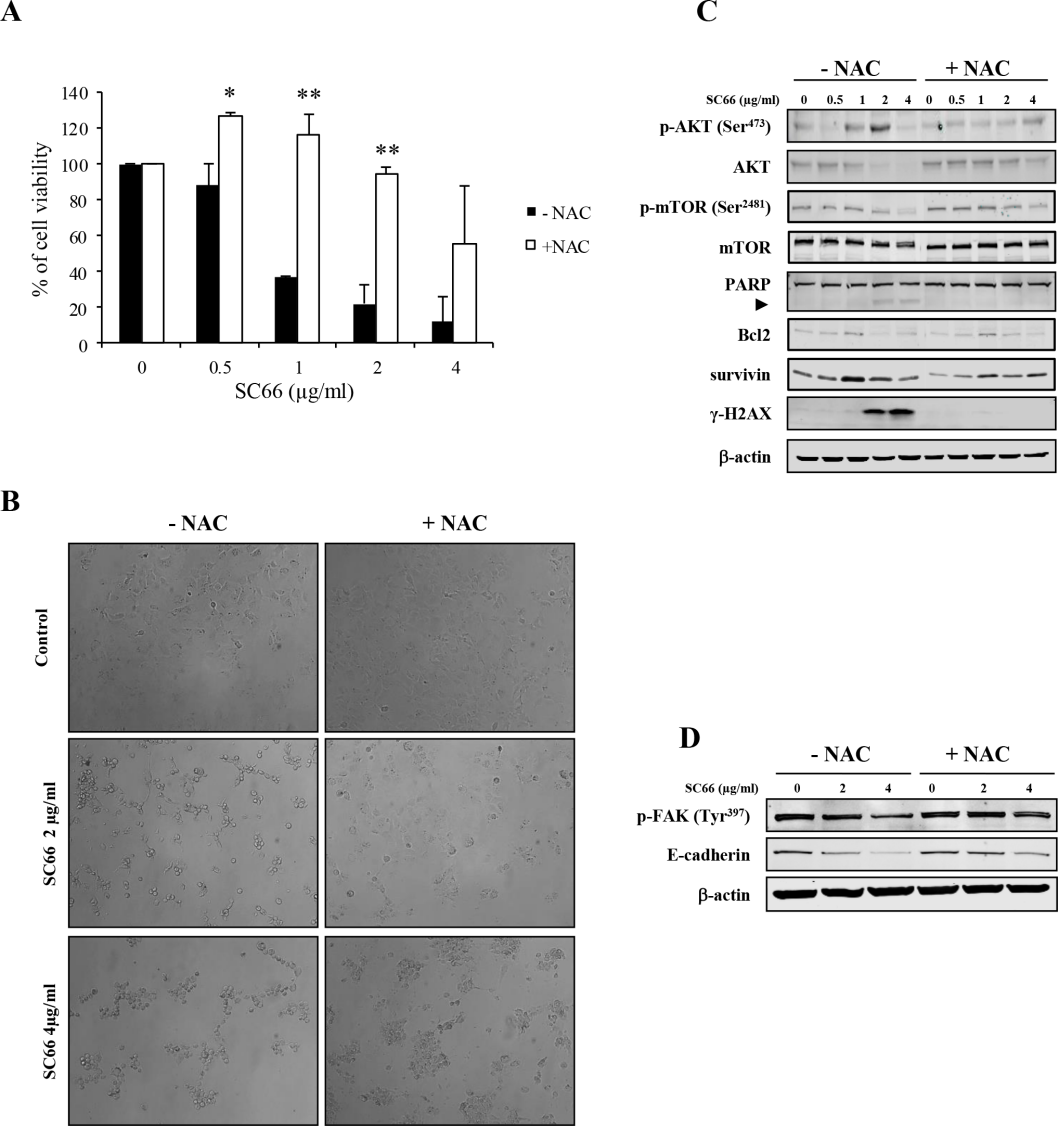
The antioxidant NAC reverts SC66-induced anoikis, cell growth and AKT signaling inhibition **(A)** Cell viability was assessed by MTS assays. Hep3B cells were pre-treated with antioxidant NAC for 2 hours and then treated with SC66 at the indicated concentrations for 24 hours. Data are expressed as the percentage of control cells and are the means ± SD of three separate experiments, each of which was performed in triplicate. *p < 0.05, **p < 0.001 versus control. **(B)** Bright field photomicrograph of Hep3B cells pre-treated with NAC (2 mM) for 2 hours and then treated with different concentrations of SC66 for 24 hours. The changes in cell morphology after SC66 treatment were reverted in the presence of NAC. **(C)** The levels of AKT, mTOR, Bcl2, survivin, γ-H2AX proteins and PARP cleavage induction were analyzed by Western blotting. Cells were treated with different concentrations of SC66 for 24 hours with or without NAC (2 mM). The data shown represent three independent experiments with comparable outcomes. The arrowhead indicates the 85 kDa form of PARP. **(D)** Phosphorylated levels of FAK and E-cadherin levels were determined by Western blotting after SC66 treatment with or without NAC (2 mM). The data shown represent two independent experiments with comparable outcomes.

We hypothesized that by inhibiting ROS effects NAC might revert the effects of SC66 on cell viability, and also inhibit SC66-induced anoikis. In order to validate this hypothesis we evaluated cell viability after SC66 treatment with and without NAC. The histograms in Figure 6A demonstrated that the presence of NAC reverted the effects of SC66 on cell viability at low drug doses, whereas it partially inhibited the effects at 4 μg/ml. At the molecular level, Western blot analysis also showed an almost complete recovery of phosphorylated and total AKT levels in Hep3B cells treated with SC66 in the presence of NAC (Figure 6C), as well as inhibition of PARP cleavage (Figure 6C).

As mentioned above, many drugs may sensitize cancer cells to anoikis through ROS generation. In the presence of NAC, a reduction in the number of rounded and floating Hep3B cells was observed, with cells spread on the plate surface even when treated with SC66, suggesting that NAC also abrogates the phenotypic effects of SC66 on cell morphology (Figure 6B).

In addition, Western blot analysis demonstrated that after treatment with SC66 in the presence of NAC both E-cadherin and FAK phosphorylation levels were almost restored (Figure 6D), therefore we can assume that anoikis was induced by SC66 via ROS production.

ROS are known to induce DNA damage, therefore we evaluated the expression of the phosphorylated form of H2AX (γ-H2AX), a well-known marker of double strand DNA breaks (DSBs). γH2AX was strongly induced after SC66 treatment and returned to the baseline levels of untreated cells after treatment with NAC (Figure 6C). These results suggested that ROS generation promotes DNA damage in Hep3B cells treated with SC66 and therefore contributes to cell death induction.

### SC66 significantly potentiates the effects of doxorubicin and everolimus in Hep3B cells

**Figure 7:**
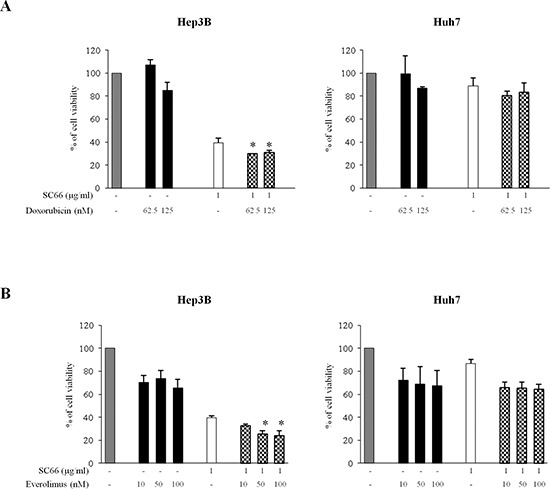
SC66 significantly reduced cell viability in combination with doxorubicin and everolimus in Hep3B cells Cell viability was assessed by MTS assays. Hep3B cells and Huh7 cells were treated with combination of SC66 and doxorubicin **(A)** or everolimus **(B)** at the indicated doses for 24 hours. Data are expressed as the percentage of control cells and are the means ± SD of three separate experiments, each of which was performed in triplicate. *p < 0.05 versus single treatments.

We next examined combination of SC66 with conventional chemotherapeutic drugs, such as doxorubicin, which is used in clinical protocols for HCC treatment, or with new target specific drugs, such as the mTOR inhibitor everolimus (also known as RAD001), currently under clinical trials in different cancer types, including HCC. We measured the cytotoxic effects after 24 hours of combination of SC66 with doxorubicin and with everolimus in both HCC cell lines using MTS assays (Figure 7). Hep3B and Huh7 cells were treated with SC66 at a single concentration (1 μg/ml) and two different concentrations of doxorubicin (62.5 and 125 nM) (Figure 7A), or three concentrations of everolimus (10-50-100 nM) (Figure 7B). In comparison with single treatments, combination treatments significantly reduced cell viability only in Hep3B cells.

### SC66 displays anticancer activity *in vivo*

**Figure 8:**
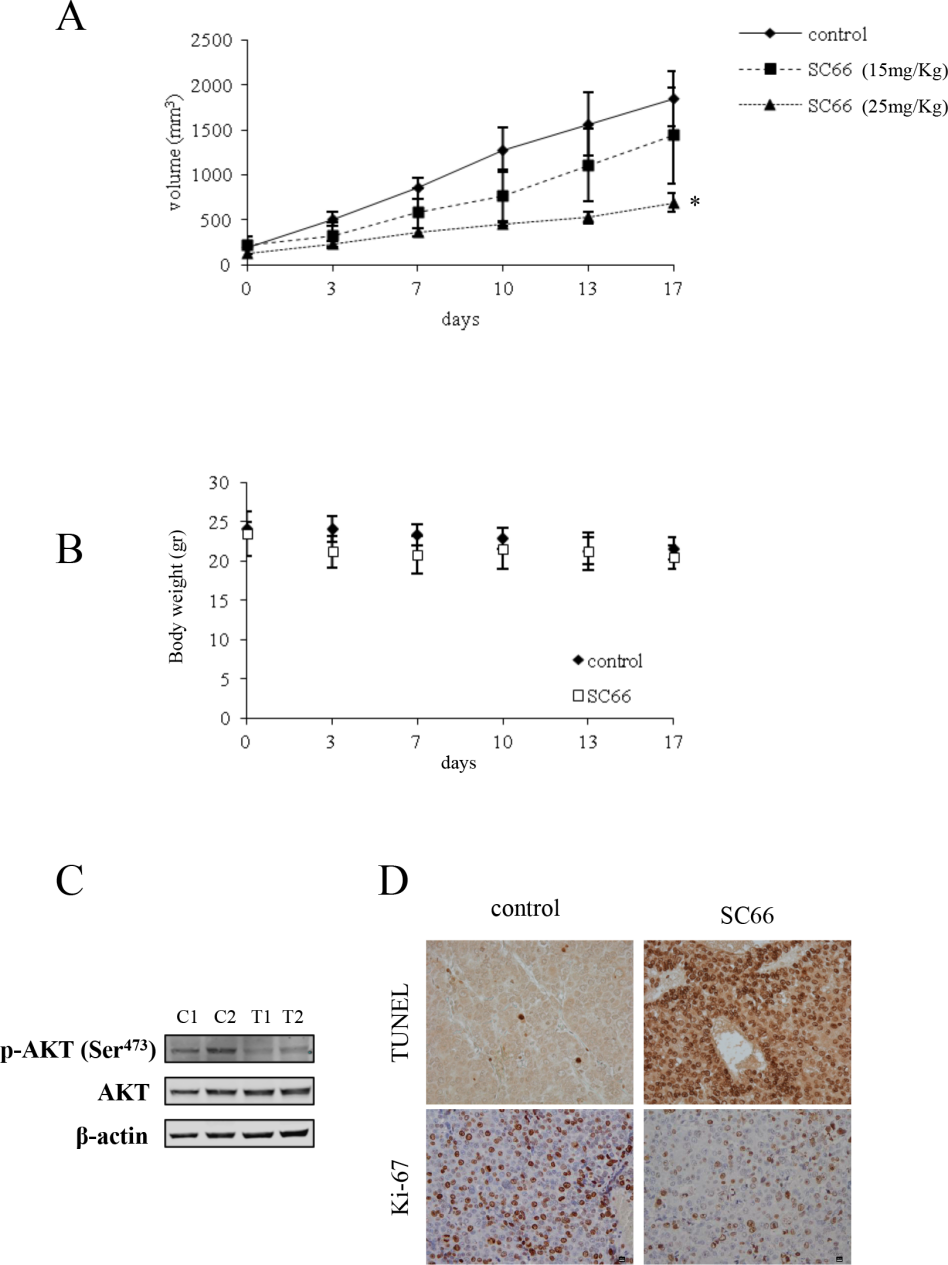
The effect of SC66 on xenograft models of Hep3B cells **(A)** Effect of SC66 on tumor growth. Once tumors were engrafted and palpable, mice (*n* = 6) were treated twice a week with SC66 at the indicated concentrations, as described in Materials and Methods. The curve of tumor growth was compared with that of control mice treated with vehicle alone, *p < 0.05. **(B)** Body weight alteration analysis. Mice treated with 25 mg/kg SC66 were weighed twice a week and the weight presented in the graphs. **(C)** Representative Western blot showing AKT and phospho-AKT levels of two mice treated with vehicle alone (control, C1 and C2) and two mice treated with 25 mg/kg SC66 (T1 and T2). **(D)** Immunohistochemical staining was performed on formalin-fixed paraffin-embedded tumor tissues. Tissue from control mice and mice treated with 25 mg/kg SC66 were stained for Ki-67 proliferation index and apoptosis (TUNEL staining).

To demonstrate the effectiveness *in vivo* of SC66 on HCC, a mouse xenograft tumor model of Hep3B cells was used. When tumors became palpable, at a size of about 150 mm^3^, mice were randomized into three groups of 6 animals each. The treated group received SC66 at 15 and 25 mg/Kg twice a week via *i.p*. injection, while the untreated group received the vehicle alone. Treatment with 25 mg/Kg SC66 significantly reduced tumor volume to 37% on day 17 when compared with tumors of the untreated group (Figure 8A). Changes in animal body weight were also monitored. As observed in Figure 8B, mice treated with 25 mg/kg SC66 did not show a significant loss of body weight when compared with mice treated with vehicle alone, suggesting a satisfactory level of drug cytotoxicity.

Western blot analysis performed on homogenates from tumor tissues of mice treated with SC66 showed lower p-AKT levels (Figure 8C) than those of untreated mice. Unexpectedly, we did not observe the reduction in total AKT levels found in *in vitro* studies. Nevertheless, immunohistochemical analysis showed an increase in the number of apoptotic cells (TUNEL assays), and a lower proliferation marker Ki-67 expression in tumor tissues from animals treated with SC66, than in the tissues of the untreated animals (Figure 8D).

## DISCUSSION

HCC is the predominant form of primary liver cancer and the third cause of cancer-related death worldwide. The survival rate for most HCC patients is extremely low, due to the lack of appropriate and effective therapy, therefore developing novel therapeutic strategies is a high priority. Considering that the PI3K/AKT/mTOR pathway is a key pathway in HCC, as its activation induces cell proliferation and increases survival and therapy resistance, in the present study we investigated the potential antitumor activity of SC66, a novel allosteric inhibitor of AKT activity, on HCC cell lines. SC66 affects AKT/mTOR signaling in HCC cells as shown by a decrease in AKT phosphorylation levels and in total protein levels. Following signaling inactivation we also found a reduction in the levels of mTOR and 4E-BP1 phosphorylation, downstream targets of AKT signaling. SC66 decreased cell growth in a dose- and time-dependent manner in all the HCC cell lines studied. However, each cell line demonstrated a different sensitivity to the drug, with Hep3B cells being the most sensitive cell line, and Huh7 cells the least responsive. The reasons for the different behavior remain elusive and the mechanism(s) of resistance to SC66 has to be elucidated. One possibility could be related to the different characteristics of differentiation, biological behavior and genetic defects of the different cell lines reflecting the heterogeneity observed in primary HCC tissues.

Inhibition of cell viability in Hep3B cells was associated with apoptosis induction, which correlated with caspase-3/7 activation, PARP cleavage and down-regulation of anti-apoptotic proteins Bcl2 and survivin.

However, the Hep3B cells did not display the classical morphological features of apoptotic cells. After treatment with SC66 in Hep3B cells, we observed strong changes in morphology causing a loss of cell contact with the extracellular matrix molecules. The cells became rounded, slightly attached to each other, similar to beads on a string, and were floating. The hypothesis that the cell death observed might be anoikis, a form of apoptosis resulting from the loss of cell adhesion to the extracellular matrix molecules [26–31], was confirmed by the following observations: first, a down-regulation of E-cadherin levels and an up-regulation of Snail (the negative regulator transcription factor of the *E-cadherin (CDH1)* gene) [32], both considered to be markers of anoikis [28–32]; second, changes in the distribution of vimentin which slightly increased and accumulated in the perinuclear zone; third, a redistribution of actin cytoskeleton as observed after staining with phalloidin; and finally, a clear reduction in the phosphorylated form of FAK, a kinase that regulates cell adhesion and acts as a signal transducer in response to integrins [37, 38]. It is worth noting that cells which became non-adherent after SC66 exposure were not able to re-adhere to the plate after the inhibitor was removed, suggesting that the death program was irreversible.

Many compounds may sensitize cancer cell to anoikis through ROS generation [33–36]. ROS have been reported to be important mediators of anoikis and are related to caspase activation [33, 36]. Recent evidence suggests that disruption of integrin contact with fibroblasts can lead to cell detachment which is preceded by a rise in intracellular ROS levels [31]. On the other hand, it has been demonstrated that detachment of endothelial cells resulted in a dramatic rise in ROS levels and, furthermore, that this rise contributed to anoikis [34]. On the basis of this evidence and our observations, we ascertained the effects of SC66 on ROS production. Measurement of ROS levels demonstrated a dose-dependent increase in ROS after treatment with SC66, suggesting that anoikis might be due to the presence of high levels of ROS, as confirmed by the reversion of SC66 effects on cell morphology, cell viability, E-cadherin and vimentin levels as well as on FAK phosphorylation observed after treatment with the antioxidant NAC. In cells co-treated with the antioxidant, an almost complete recovery of phosphorylated and total AKT was also observed, as well as inhibition of PARP cleavage. Finally, levels of γH2AX, known to be a marker of DNA damage, were strongly increased after SC66 treatment and levels in treated cells returned to baseline after treatment with NAC.

Interestingly, SC66 significantly potentiates the effects of both conventional chemotherapeutic drug (doxorubicin) and molecularly targeted anticancer agent (everolimus), offering new opportunity for treatment of HCC.

In summary, our *in vitro* results demonstrated for the first time that SC66 affects HCC cell growth and this was due to the inhibition of AKT pathways and ROS induction, and that both of these contributed to HCC cell death. Furthermore, the antitumor effects of SC66 were confirmed *in vivo*. In the mouse xenograft tumor model of Hep3B cells, SC66 treatment significantly reduced tumor volume to 37% on day 17 of treatment when compared with tumors in the untreated group. The inhibition of cell growth correlated with a reduction in phospho-AKT levels in the tumors of animals treated with SC66. However, unexpectedly, we did not observe the reduction in total AKT levels observed *in vitro*, and the reason for this result is not understood. Nevertheless, immunohistochemistry analysis revealed a reduction in cell proliferation markers and a higher degree of apoptosis in tumor tissues examined from xenograft mice treated with SC66 than in controls.

Overall, our results for the first time highlight the potential of the novel AKT inhibitor SC66 as a therapeutic drug for HCC treatment, providing the rationale for future experimental use in humans.

## MATERIALS AND METHODS

### Cell lines, cell culture and reagents

The human hepatocarcinoma cell lines HepG2, Huh7, PLC/PRF/5, Hep3B and HA22T/VGH used in this study had a low passage number and were maintained in RPMI medium (SIGMA, Milan, Italy), containing 10% (v/v) Fetal Bovine Serum (FBS) (GIBCO, Life Technologies, Monza MB, Italy). HepG2, Huh-7 and HA22T/VGH cells used in this study were a gift from Prof. M. Levrero (Department of Internal Medicine, Sapienza University, Rome, Italy). Hep3B and PLC/PRF/5 cells used in this study were a gift from Prof. O. Bussolati (Unit of General and Clinical Pathology, Department of Experimental Medicine, University of Parma, Parma, Italy). The HCC cell lines have different characteristics of differentiation, biological behavior and genetic defects [39]. SC66 was purchased from Cayman Chemicals (Ann Arbor, MI, USA). Doxorubicin and everolimus (RAD001) were purchased from SIGMA.

### Cell viability assays

Cells (5 × 10^3^/well) were distributed into each well of 96-well microtiter plates and then incubated overnight. At time 0, the medium was replaced with fresh complete medium and different doses of SC66 were added. Cells were cultured for 24, 48 and 72 hours. At the end of treatment, MTS assays were performed using the CellTiter Aqueous OneSolution kit (Promega Corporation, Madison, WI, USA) according to the manufacturer's instructions. Cell viability was expressed as a percentage of the absorbance measured in the control cells. Values were expressed as means ± SD of three separate experiments, each performed in triplicate. In recovery experiments, cells were pre-treated with the antioxidant N-Acetyl-cysteine (NAC) (SIGMA) for 2 hours and then the drug was added.

### Caspase activity assays

Cells (2 × 10^4^/well) were treated with 2 and 4 μg/ml SC66 and after 1, 3 and 6 hours the levels of caspase3/7 activities in the cells were measured by the Caspase-Glo^®^ 3/7 Assay (Promega) according to the manufacturer's instructions. Results were expressed as relative luminescence units (RLU). Values were the mean ± SD of two separate experiments, each performed in duplicate.

### Western blot analysis

Whole cellular lysates from 0.5 × 10^6^ cells were obtained using RIPA buffer (Cell Signaling Technologies Inc., Beverly, MA, USA) and Western blots were performed using the methodology for the Odyssey® infrared imaging system (LI-COR Biosciences, NE, USA). After transfer nitrocellulose membranes were placed in Odyssey® blocking buffer (OBB, LI-COR) diluted in tris-buffered saline (TBS) and incubated for 1 hour at room temperature. Primary antibodies were diluted in OBB. Secondary antibodies conjugated to IRDye® 800CW (LI-COR) or Alexa Fluor 680 (Molecular Probes, Invitrogen Carlsbad, CA, USA) were diluted in OBB. Membranes were scanned and analyzed with an Odyssey IR scanner using Odyssey 3.0 imaging software. Antibody signals were analyzed as integrated intensities of regions defined around the bands of interest in either channel, with primary antibodies raised against survivin (Abcam Limited, Cambridge, UK), β-actin (SIGMA), phospho-AKT, AKT, phospho-mTOR, mTOR, phospho-4E-BP1, 4E-BP1, PARP and Bcl2 (Cell Signaling Technologies), phospho-FAK, E-cadherin and β-catenin (BD Transduction Laboratories, Lexington, KY, USA), Snail (Santa Cruz, Dallas, Texas, USA).

### Flow cytometry analysis

After 24 hours of SC66 treatment, 1 × 10^6^ cells were collected and stained with propidium iodide (PI) (Invitrogen) and the percentage of apoptotic cells was determined as previously described [39].

### Measurement of reactive oxygen species (ROS)

The intracellular accumulation of ROS was determined using the fluorescent probe 2′,7′-difluorodihydrofluorescein diacetate (H_2_DCFDA) (Invitrogen). Cells (2 × 10^5^) were treated with various concentrations of SC66 for the indicated time and then incubated with the probe (1 μM) in the dark at 37°C, 5% CO_2_ for 15 min. Cells were observed with fluorescence microscopy (Leica GmbH, Germany) and photographed.

### TUNEL assays

Cells (2 × 10^4^/well) were cultured in 8-well chamber slides overnight. After treatment for 24 hours with various concentrations of SC66, cells were washed twice with PBS and fixed in 4% paraformaldehyde solution for 25 min at room temperature. Apoptotic cells were detected by terminal deoxynucleotidyl transferase-mediated dUTP nick end-labeling (TUNEL) assay using the DeadEnd^TM^ Colorimetric TUNEL System Kit from Promega, following the manufacturer's instructions. Cells were visualized with an Axioskop microscope (Zeiss, Oberkochen, Germany).

### Colony formation assays

Cells (1.0 × 103/well) were plated in six-well plates in growth medium, and after overnight attachment cells were exposed either to SC66 or vehicle for 48 hours. The cells were then washed and allowed to grow for 14 days in drug-free conditions, after which the cell colonies were fixed with 70% ethanol at 4°C for 20 min and stained with crystal violet (0.1% in H_2_O) for 5 min. The plates were rinsed with water, air-dried, photographed and evaluated for colony estimation. Colonies containing more than 50 cells were counted. All experiments were performed in duplicate and repeated twice.

### Small interfering RNA (siRNA) transfection

Hep3B cells (3 × 10^5^) were cultured in 6-well plates in 2 ml of RPMI medium in 10% FBS. After 24 hours the cells at 30–50% confluences were transfected in serum-free medium with pre-validated human AKT siRNA (Qiagen, Germantown, MD, US) or control (scrambled) siRNA (Qiagen) at a final concentration of 50 nM, using RNAiMAX (Invitrogen, Carlsbad, CA) following the manufacturer's instructions. 24 hours after transfection cells were trypsinized, counted and plated in 96-well plates for MTS assay and in 3 cm diameter Petri dishes for protein extraction. All experiments were performed in triplicate and repeated twice.

### Immunofluorescence staining

Cells (2 × 10^5^/well) were cultured in 8-well chamber slides. After 24 hours cells were treated with SC66, after which they were fixed in 3.7% paraformaldehyde for 15 minutes, and permealized using 0.25% Triton-X-100 for 5 minutes at room temperature. Cells were then incubated for 1 hour with monoclonal anti-vimentin antibody (Santa Cruz) in 1% bovine serum albumin (BSA) in PBS at room temperature in a moist chamber. Immunofluorescence staining was obtained by incubation for 1 hour with secondary antibodies conjugated to Alexa Fluor 555 (Invitrogen). Actin staining was performed using phalloidin (SIGMA) and incubated with cells for 30 min at room temperature. Cells were counterstained with DAPI, mounted on slides and examined with fluorescence microscope (Zeiss).

### *In vivo* studies

Male nude athymic mice (Fox1 nu/nu) aged 4 weeks were obtained from Harlan (Udine, Italy) and allowed to acclimatize for 1 week. Suspensions of 10 × 10^6^ Hep3B cells in 0.2 ml of PBS were inoculated into the right flank of the animal. When tumors became palpable (around 150 mm^3^), the mice were randomly divided into three groups of 6 animals each, with the various tumor volumes equally distributed among the three groups. Two groups of mice were treated twice a week with 15 and 25 mg/Kg SC66 suspended in DMSO, further diluted in a solution of 25% ethanol and administered via *i.p*. injection. The control group received the vehicle alone. Tumor volumes were determined twice a week using calipers. Primary tumor volumes were calculated with the formula: *v* = length × (width)^2^/2. Mice were euthanized by cervical dislocation when the tumor burden exceeded 10% of animal body weight, or when tumor ulcerated or other conditions of morbidity were ascertained, in conformity with institutional guidelines which are in compliance with national (D.L., 116 G.U., Suppl.40; 18 February 1992) and international laws and policies (ECC Council Directive 86/609, OJ L358.1, 12 December 1987). This study was authorized by the Italian Ministry of Health (D.M. n. 39/2014-B).

### Immunohistochemistry (IHC) analyses

Immunohistochemical studies were performed on formalin-fixed paraffin-embedded tumor tissues. Serial sections (4 μm thick) on glass slides were washed in xylene and hydrated in decreasing concentrations of alcohol. For antigen retrieval the slides were heated in sodium citrate solution (pH 6.0) at 96°C for 20 min. Endogenous peroxidase activity was quenched with 3% hydrogen peroxide in methanol for 30 min. Then the slides were treated with 1% BSA for 30 min and incubated for 1 hour at room temperature in the presence of 0.1% BSA with a monoclonal rabbit antibody for nuclear antigen, Ki-67 (1:100) (Biocare Medical, CRM325). At the end, the sections were treated for 30 min with secondary biotinylated immunoglobulin anti-rabbit antibody (DAKO, LSAB Kit, K0690, Denmark). The sections were then incubated with a streptavidin-horseradish peroxidase conjugate for 1 hour, followed by chromogen 3-3′ diaminobenzidine tetrahydrochloride for 1 min, and counterstained with Mayer's hematoxylin. The specific primary antibody was replaced with PBS in tissue sections used as negative controls. All immunostained sections were analyzed at a × 400 magnification (five fields). Images of stained slides were captured using Leica DMR microscope equipped with a Leica DFC 320 digital camera.

Apoptotic cells were detected by TUNEL assay using the DeadEnd^TM^ Colorimetric TUNEL System Kit from Promega, following the manufacturer's instructions. Cells were visualized with an Axioskop microscope (Zeiss).

### Statistical analysis

Statistical analysis was performed using Student's two-tailed *t* test. Statistical significance was *p* < 0.05.
